# A regionally scalable habitat typology for assessing benthic habitats and fish communities: Application to New Caledonia reefs and lagoons

**DOI:** 10.1002/ece3.6405

**Published:** 2020-06-08

**Authors:** Dominique Pelletier, Nazha Selmaoui‐Folcher, Thomas Bockel, Thomas Schohn

**Affiliations:** ^1^ Ifremer LEAD Nouméa Nouvelle‐Calédonie France; ^2^ Ifremer EMH Nantes France; ^3^ Université de la Nouvelle‐Calédonie ISEA Nouméa Nouvelle‐Calédonie France

**Keywords:** Coral Sea Marine Park, data mining, habitat prediction, habitat typology, in situ monitoring, marine protected areas, scaling up, supervised classification rules, underwater video

## Abstract

Scalable assessments of biodiversity are required to successfully and adaptively manage coastal ecosystems. Assessments must account for habitat variations at multiple spatial scales, including the small scales (<100 m) at which biotic and abiotic habitat components structure the distribution of fauna, including fishes. Associated challenges include achieving consistent habitat descriptions and upscaling from in situ‐monitored stations to larger scales.

We developed a methodology for (a) determining habitat types consistent across scales within large management units, (b) characterizing heterogeneities within each habitat, and (c) predicting habitat from new survey data. It relies on clustering techniques and supervised classification rules and was applied to a set of 3,145 underwater video observations of fish and benthic habitats collected in all reef and lagoon habitats around New Caledonia.

A baseline habitat typology was established with five habitat types clearly characterized by abiotic and biotic attributes. In a complex mosaic of habitats, habitat type is an indispensable covariate for explaining spatial variations in fish communities. Habitat types were further described by 26 rules capturing the range of habitat features encountered. Rules provided intuitive habitat descriptions and predicted habitat type for new monitoring observations, both straightforwardly and with known confidence. Images are convenient for interacting with managers and stakeholders.

Our scheme is (a) consistent at the scale of New Caledonia reefs and lagoons (1.4 million km^2^) and (b) ubiquitous by providing data in all habitats, for example, showcasing a substantial fish abundance in rarely monitored soft‐bottom habitats. Both features must be part of an ecosystem‐based monitoring strategy relevant for management.

This is the first study applying data mining techniques to in situ measurements to characterize coastal habitats over regional‐scale management areas. This approach can be applied to other types of observations and other ecosystems to characterize and predict local ecological assets for assessments at larger scales.

## Graphical abstract

A methodology producing a scalable habitat proxy for ecological assessment and predicting habitat with a high confidence level. Applied to a unique set of in situ video monitoring observations, entailing image‐based display of habitats for science uptake. First application of supervised classification rules in ecology.

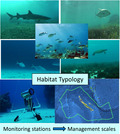

## 1 INTRODUCTION

Assessing the ecological status of ecosystems and natural resources in the face of anthropogenic and environmental stressors is necessary to inform and guide appropriate management decisions (Mumby & Steneck, 2008). Consistently with an ecosystem‐based (EB) approach to management (Long, Charles, & Stephenson, 2015), assessments of biodiversity and resource status are necessary at the scale of large spatial entities such as territories or regional ecosystems. In this paper, assessment refers to periodic evaluation of changes in monitoring‐based indicators of biodiversity linked to management targets, for example, for marine protected areas (MPA) (Hockings, Stolton, Leverington, Dudley, & Courrau, 2006). However, the spatial and temporal distribution of biodiversity indicators depends on both management‐related factors (anthropogenic pressures and/or protected area status) and environmental factors, such as habitat, which must thus be accounted for in monitoring and assessment. It has long been acknowledged that the spatial distribution of natural communities is largely shaped by the characteristics and availability of their habitat in the environment (Bell, McCoy, & Mushinsky, 1991). Shallow marine ecosystems typically encompass a variety of habitats determined by biological and physical features, such as benthic cover, depth, wave exposure, and modified by anthropogenic pressures such as fishing or pollution. Habitat features strongly influence the structure of demersal–benthic fish communities (Anderson & Millar, 2004). This influence occurs at a range of spatial scales (Bach, Saunders, Newman, Holmes, & Harvey, 2019; García‐Charton & Pérez‐Ruzafa, 2001) from small (<100 m) to larger (>100 m) (see, e.g., Grober‐Dunsmore et al. (2008) for references); even at meter scale, habitat variations influence spatial patterns of fishes and other macrofauna (e.g., Brokovich, Baranes, & Goren, 2006; Ferraris, Pelletier, Kulbicki, & Chauvet, 2005; García‐Charton & Pérez‐Ruzafa, 2001; Gratwicke & Speight, 2005; Komyakova, Jones, & Munday, 2018). The influence of habitat on shallow fishes has been studied mostly in either rocky habitats (Quaas, Harasti, Gaston, Platell, & Fulton, 2019; Smith & Anderson, 2016; Teixeira‐Neves, Neves, & Araújo, 2015) or soft‐bottom areas (van Lier, Harasti, Laird, Noble, & Fulton, 2017), but not over all habitats in a given area. Yet, fish are connected to multiple habitats via ontogenic migrations, larval dispersal, and daily movement (Perry, Staveley, & Gullström, 2018), meaning that from an EB perspective, all habitats within the concerned ecosystem should be considered when assessing coastal fish communities.

This paper focuses on benthic coastal habitats described by geometric parameters, for example, complexity, rugosity (Charbonnel, Ruitton, Serre, Harmelin, & Jensen, 2002), and other measures of configuration or landscape metrics (Grober‐Dunsmore et al., 2008), geomorphology (e.g., Andréfouët & Torres‐Pullizza, 2004), and biotic and abiotic covers. Small‐scale (<100 m) patchiness of habitats is preferably captured by in situ measurements. Here, we characterize benthic habitats at observation scale using panoramic underwater video. Measurements of habitats and fish communities were collected on both hard substrates and soft‐bottom areas within vast marine managed areas where periodic assessment of both habitats and fish communities is required.

To be utilized as an explanatory factor in assessments, a concise description of habitat is needed at each station. In the past, habitat typologies (also termed systematic classification schemes; Mumby and Harborne (1999)) have been obtained from quadrat and distance‐based transect data using nonsupervised multivariate methods such as factorial and cluster analyses (Ferraris et al., 2005; Mumby & Harborne, 1999; Pelletier et al., 2012). The cluster index forms a concise habitat proxy (covariate) for explaining spatial variations of fish assemblages (Ferraris et al., 2005) or for informing management and science through standardized maps. Yet, this synthetic proxy neglects within‐habitat heterogeneity, which also influences spatial variations of macrofauna (see above). In addition, predicting habitat from data collected either in follow‐up monitoring surveys or at other locations is tedious as it requires mathematical computations, namely projecting the new data on the clusters.

In the case of large databases, mining techniques are an appropriate and efficient way to determine meaningful association rules between variables of interest under the form of sets of conditions on their values, along with measures of confidence and frequency (Agrawal, Imielinski, & Swami, 1993; Fournier‐Viger, Wu, & Tseng, 2012; Han, Pei, Yin, & Mao, 2004). Significant rules are typically frequent patterns encountered in the data set at hand (Han et al., 2004), but methods are also developed for mining rare patterns (Piri, Delen, Liu, & Paiva, 2018).

Using both clustering techniques and supervised classification rules, we developed a methodology for (a) devising a habitat typology consistent across scales within large management units; (b) characterizing heterogeneities within each habitat type; and (c) predicting habitat from new survey data. The methodology was applied to a comprehensive data set of underwater video observations collected in New Caledonia (NC, Southwest Pacific).

## 2 MATERIALS AND METHODS

### 2.1 Study area

**FIGURE 1:**
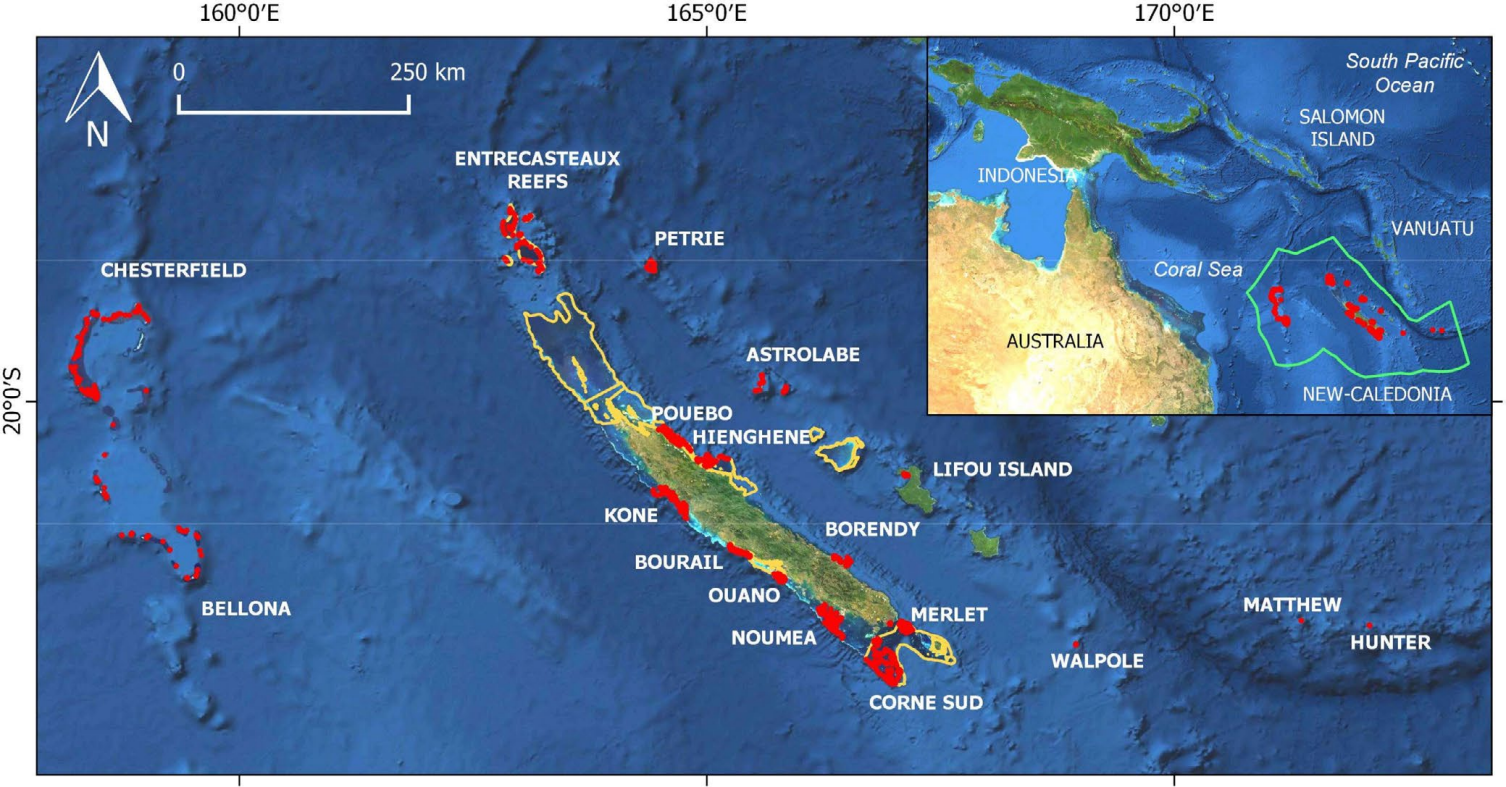
Study area showing distribution of 3,145 sampling stations (red). Inset: location of NC in the southwest Pacific, with the perimeter of the EEZ and external boundary of the Coral Sea Marine Park (CSMP) in green. The CSMP coastal boundary is the barrier reef surrounding the main island and the three islands of the Loyalty archipelago (including Lifou) located between Astrolabe and Walpole. Boundaries of the World Heritage property are in orange

The study area encompasses NC reef and lagoon areas (southwest Pacific Ocean, 17–24° S, 158–172° W; Figure 1). NC comprises the Loyalty archipelago and a main island, approximately 400 km long and 50 km wide, surrounded by a large lagoon subject to a range of anthropogenic pressures, particularly close to Noumea City. Outside of the lagoon, the NC Exclusive Economic Zone (EEZ) comprises remote well‐preserved reefs, islands, and atolls that make up for the Coral Sea Marine Park (CSMP, 1,300,000 km^2^) declared in 2014 (Figure 1). Aside from CSMP, 15,743 km^2^ (i.e., 80%) reef and lagoon areas were declared a World Heritage (WH) serial property in 2008 due to the exceptionally high diversity of their coral reef ecosystems (https://whc.unesco.org/en/list/1115). Both WH and CSMP management involve periodic monitoring for assessment and reporting on fish resources and biodiversity.

### 2.2 Data collection

#### 2.2.1 Observation equipment

Data for benthic habitat and fishes were collected using a remote unbaited rotating underwater video system (STAVIRO; Pelletier et al., 2012). A standardized procedure for sampling design, field operations, image annotation, and data analysis was described in Pelletier, Carpentier, Roman, and Bockel (2016). The STAVIRO system consists of an HD video camera and a motor programmed to rotate the camera housing by 60° every 30 s (1 rotation ~ 3 min), yielding 6 contiguous fixed frames per 360° rotation. This relatively lightweight (6 kg) system was dropped from the boat at the station location and set horizontally on the sea bed. The system was left for 15–20 min to record the video over three complete undisturbed rotations.

#### 2.2.2 Sampling design

Stations were located at eighteen sites representative of NC coral reef areas: remote sites in the CSMP (Entrecasteaux, Petrie, Astrolabe, Chesterfield, Bellona, Matthew, Hunter, Walpole), and others around the main island (Nouméa, Koné, Pouebo, Hienghène, Bourail, Borendy, Merlet, Corne Sud and Ouano) and in the Loyalty Is. (Lifou) (Figure 1). Data were collected between 2007 and 2015, between March and September, outside of the summer season. The sampling design at each site was stratified using geomorphological maps (Andréfouët & Torres‐Pullizza, 2004) and included main reef areas and associated soft‐bottom habitats. Within each stratum, stations were distributed to cover the entire site area and account for management status (marine protected area (MPA), WH property, unprotected areas). In total, 3,145 stations were sampled (Figure 1) at depths ranging between 1 and 41 m.

#### 2.2.3 Data validation and image analysis

After fieldwork, video footage was validated when (a) underwater visibility (estimated from reference images; see below) was at least 5 m, and (b) the field of view was not obstructed by any sea floor or benthos relief that would prevent image analysis within a 5‐m radius around the system. For each valid video, habitat attributes (Table 1) were evaluated from a single rotation for an estimated 5‐m radius around the video system, corresponding to an observed surface area of ca. 78.5 m^2^. Each attribute was evaluated in each frame, and values were then averaged over the six frames of the rotation.

**TABLE 1:** Habitat attributes annotated in video footages

Attribute (parameter type)	Definition
Depth (m)	Measured from a depth gauge on the STAVIRO
Topography	Seabed steepness. If h denotes the largest altitude between troughs and elevations: h negligible, h < 1 m, 1 < h < 2 m, 2 < h < 3 m, h > 3 m
Complexity	Number and diversity in size of potential refuges: none, low, medium, strong, outstanding
Substrate	PC of five substrate categories: (a) sand; (b) debris (<0.3 m); (c) boulder (between 0.3 m and 1 m); (d) rock (>1 m); and (e) slab
Live coral	PC of live coral
Dead coral	PC of recently dead coral
Macroalgae	PC of macroalgae
Seagrass	PC of seagrass
Auxiliary attributes	
Coral form	PC of live coral per morphotype: branch, massive, digitate, foliate, others
Macroalgae	PC of erect algae and other algae
Seagrass	PC of erect and short seagrass, percent covers of seagrass per density category: dense, semidense, sparse

Note

Topography and complexity scores range between 1 and 5. Percent covers (PC) refer to the observed surface area. “Macroalgae” does not include encrusting algae. “Other algae” mostly includes algal turf, that is, typically low‐lying (mm to cm tall) layer of algae (Connell et al., 2014). “Dead coral” still retains a coral shape. Habitat annotation was derived from Clua et al. (1996).

Fish and other marine animals were identified at the most precise taxonomic level based on a reference species list, and counted on each frame and for each of three undisturbed rotations within a 5‐m radius around the system. The reference list included 42 families (Appendix 1). For each species at each station, abundance was calculated as the mean count over three rotations, which averaged out the variability between rotations. Abundances were expressed in densities as numbers of individuals per 100 m^2^ (ind/100 m^2^). Species richness was the number of species observed within a 5‐m radius around the camera during the three rotations.

Estimation of visibility, attributes, and 5‐m radius followed training of annotators with reference images comprising bright and dark fish silhouettes of several sizes filmed at a range of distances and in several visibility conditions. Training was validated after successful joint analyses of a set of images were conducted with an expert.

### 2.3 Data analysis

**FIGURE 2:**
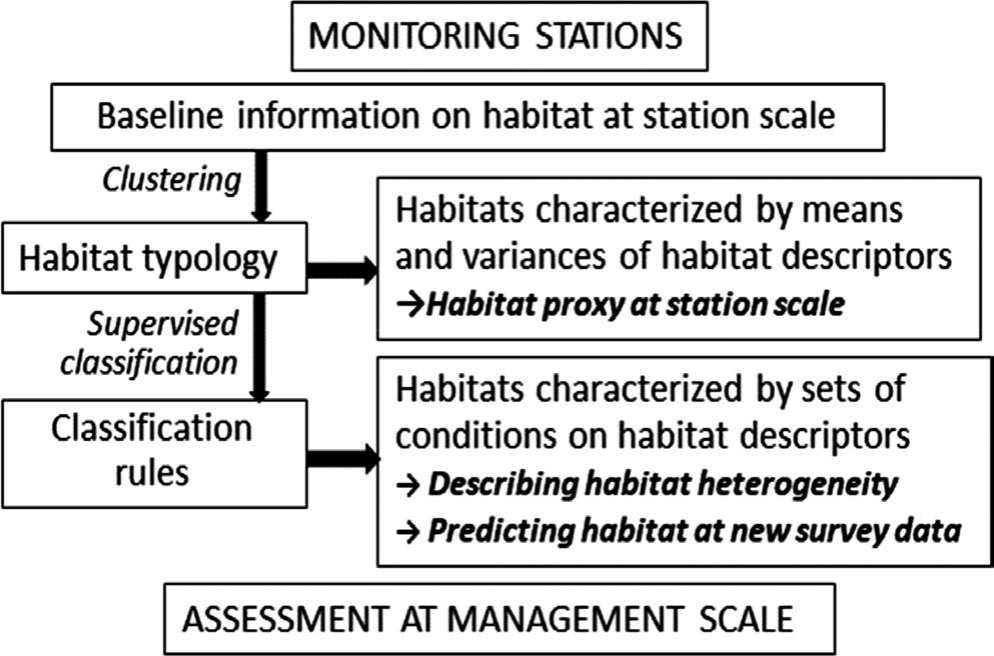
Analytical workflow: methods (left) and outputs (right)

Our classification method had two steps: (a) producing the habitat proxy (cluster index) summarizing habitat attributes at each station, and (b) deriving classification rules for describing within‐cluster heterogeneity and predicting habitat (Figure 2).

#### 2.3.1 Constructing the typology and the habitat proxy

In this broadly distributed data set, biotic covers differed strikingly between well‐preserved remote sites, and coastal sites subject to anthropogenic pressures. The typology was constructed from the 2,609 coastal stations only, and the remote stations were a posteriori projected on the typology to avoid: (a) a systematic contrast of remote and coastal stations due to average differences in live coral cover; and (b) failure to discriminate between habitat variations between coastal areas. The clusters of coastal stations were obtained by combining principal component analysis, hierarchical ascending clustering, and Random Forest (RF) modeling (Breiman, 2001, Appendix 2). Based on this typology of coastal stations, habitat was predicted at the 536 remote stations using a second RF model (Appendix 2). Clusters were characterized by habitat attributes by testing differences in means between each cluster and the overall set of stations (Pelletier & Ferraris, 2000).

The distribution of the habitat proxy was mapped at the scale of the entire territory and at the site scale. The relevance of the clusters as a habitat proxy for explaining spatial variations of fish communities was illustrated by: (a) testing the effect of habitat for two widely used metrics, overall fish abundance and species richness; and (b) computing and plotting frequency per family in each habitat.

#### 2.3.2 Classification rules

Classification rules are used to describe multivariate data sets (Appendix 3). In this paper, a classification rule is made of a set of conditions on habitat attributes that imply a specific habitat (here, the habitat proxy).

Because of the large number of possible combinations of conditions on habitat attributes, objective constraints were set to select the most interesting and relevant rules: (a) The rule comprises 3 conditions or less on habitat attributes; (b) a maximum support (number of observations satisfying the rule), and (c) a minimum confidence (proportion of observations satisfying the conditions and belonging to that habitat) (Appendix 3). Rules were extracted using the TopKRules algorithm, which retains the K rules with maximum support and a minimum confidence (*min_conf*). The algorithm was implemented using the SPMF software (Fournier‐Viger et al., 2012). Top1000 rules were searched for three *min_conf* values, 80%, 90%, and 95%, producing three sets of 1,000 rules.

The Top1000 rulesets were then selected and reorganized based on expert knowledge, to achieve a compromise between representativeness (i.e., a large proportion of the stations in each habitat were described by the rules with a high confidence level) and parsimony (not having too many rules). Each rule had to (a) include a condition on the archetypical attribute of each habitat; (b) comprise up to four conditions on habitat attributes; and (c) not overlap with another rule.

Expert knowledge was also useful to identify specific habitat attributes that were relevant to describe within‐habitat heterogeneity. Including a condition on such an attribute in some rules increased the rules' confidence by making it more specific of the habitat type. In some habitats, rules with lower confidence were considered to increase their support. The resulting set of expert‐selected rules was then used for describing within‐habitat heterogeneity. We then assessed the ability of this set of rules to predict habitat considering the confidence level for each habitat type and over all habitats.

## 3 RESULTS

### 3.1 Habitat typology and proxy

Five clusters (i.e., habitat types) were retained, each clearly characterized by an archetypical attribute and named accordingly. Three habitats pertained to soft sand‐dominated bottoms (Macroalgae, Seagrass, Sandy), while two habitats corresponded to dominant hard substrates (Live Coral and Debris). In each cluster, the archetypical attribute was larger than 15%, but for the Live Coral habitat, 113 stations displaying a lower live coral cover were assigned because they also had a substantial dead coral cover. They were set aside from the coastal station data set, which was then used to train a RF classification model (based on 1,000 trees, out‐of‐bag (OOB) error of 3.9%). From this model, habitat was predicted for the 113 stations: Respectively 77 and 35 stations were classified in the Debris and Sandy habitats, and one in the Live Coral habitat (live coral cover, 14.9%).

The second RF model trained from this consolidated typology (based on 1,000 trees, out‐of‐bag (OOB) error of 4.1%) served to predict habitat for the 536 oceanic remote stations. These were assigned to the Live Coral (48%), Sandy (27%), and Debris (25%) habitats.

The final clusters with all the stations were described by habitat attributes (Table 2, Appendix 4). Average live coral cover was unsurprisingly higher at remote stations than at coastal stations, in particular in the Live Coral habitat (Appendix 4), and two thirds of stations with live coral cover > 80% were in remote sites. In other habitats, live coral cover was sometimes high, for example, in Sandy habitat due to the presence of coral patches. Seagrass and Macroalgae habitats appeared characteristic of coastal areas where high seagrass and macroalgae covers were also observed in other habitats, thereby illustrating the heterogeneity inherent to each habitat.

**TABLE 2:** Description of habitat clusters

Habitat (# stations)	Significant habitat attributes by decreasing significance		Illustration
	Higher mean in cluster	Lower mean in cluster	
Seagrass (340)	**Seagrass**, **sand**, depth, macroalgae	**Complexity**, **topography**, live coral, dead coral, debris, slab, boulder, rock	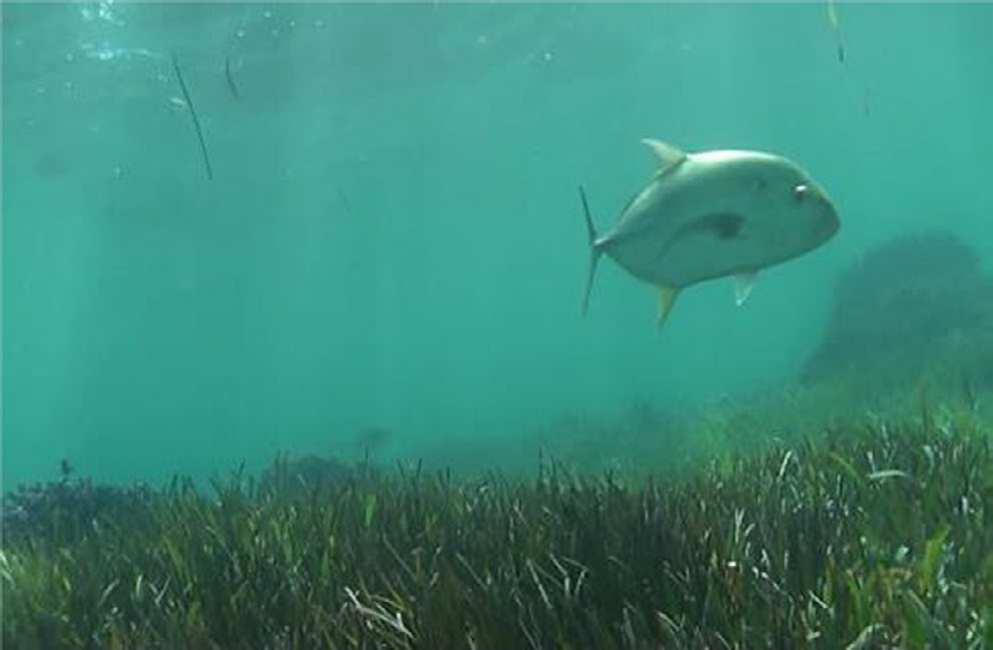
Macroalgae (175)	**Macroalgae**, sand, depth	Topography, live coral, dead coral, complexity, slab, debris, boulder, rock	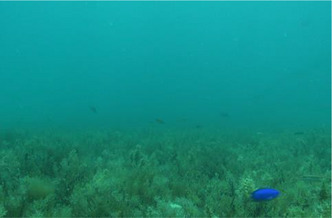
Sandy (1,157)	**Sand**	**Complexity, live coral, topography**, seagrass, slab, debris, dead coral, macroalgae, boulder, depth, rock	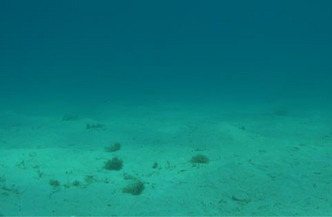
Debris (756)	**Debris, slab, boulder,** rock, complexity, dead coral, topography	**Sand,** seagrass, live coral, macroalgae, depth	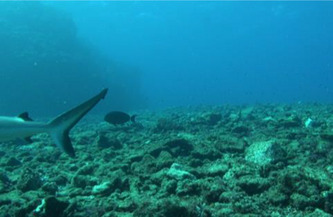
Live Coral (717)	**Live coral**, **complexity, topography, dead coral**, depth	**Sand,** seagrass, debris, macroalgae, boulder, rock	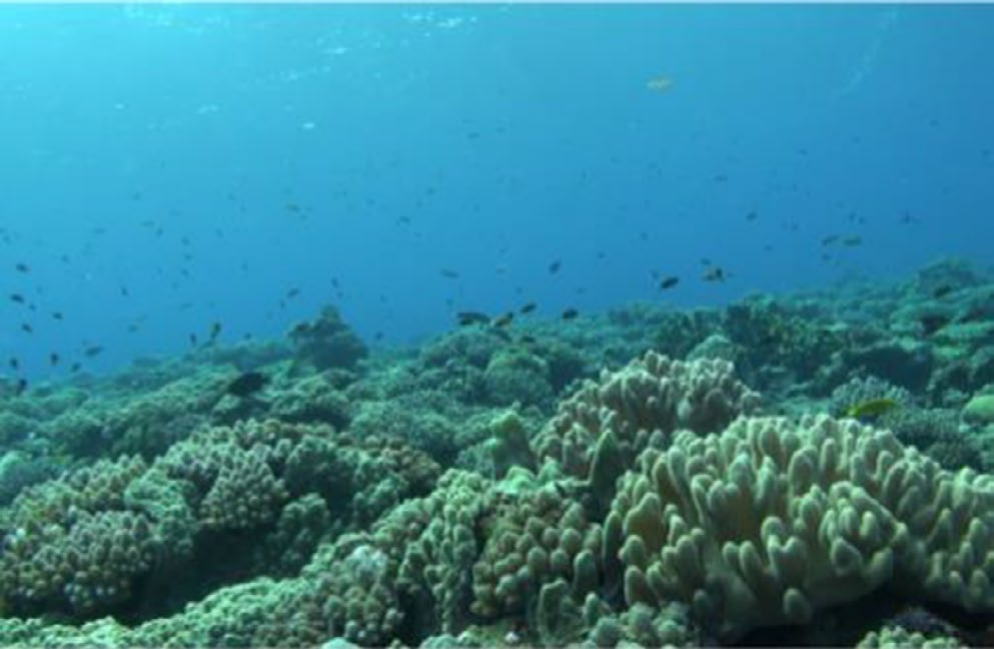

Note

Highly significant attributes (*p* < 10^–50^) are in bold. Higher (resp. lower) mean in cluster signifies that the mean attribute was higher (resp. lower) in the cluster than on average over all stations (statistics and boxplots in Appendix 4).

**FIGURE 3:**
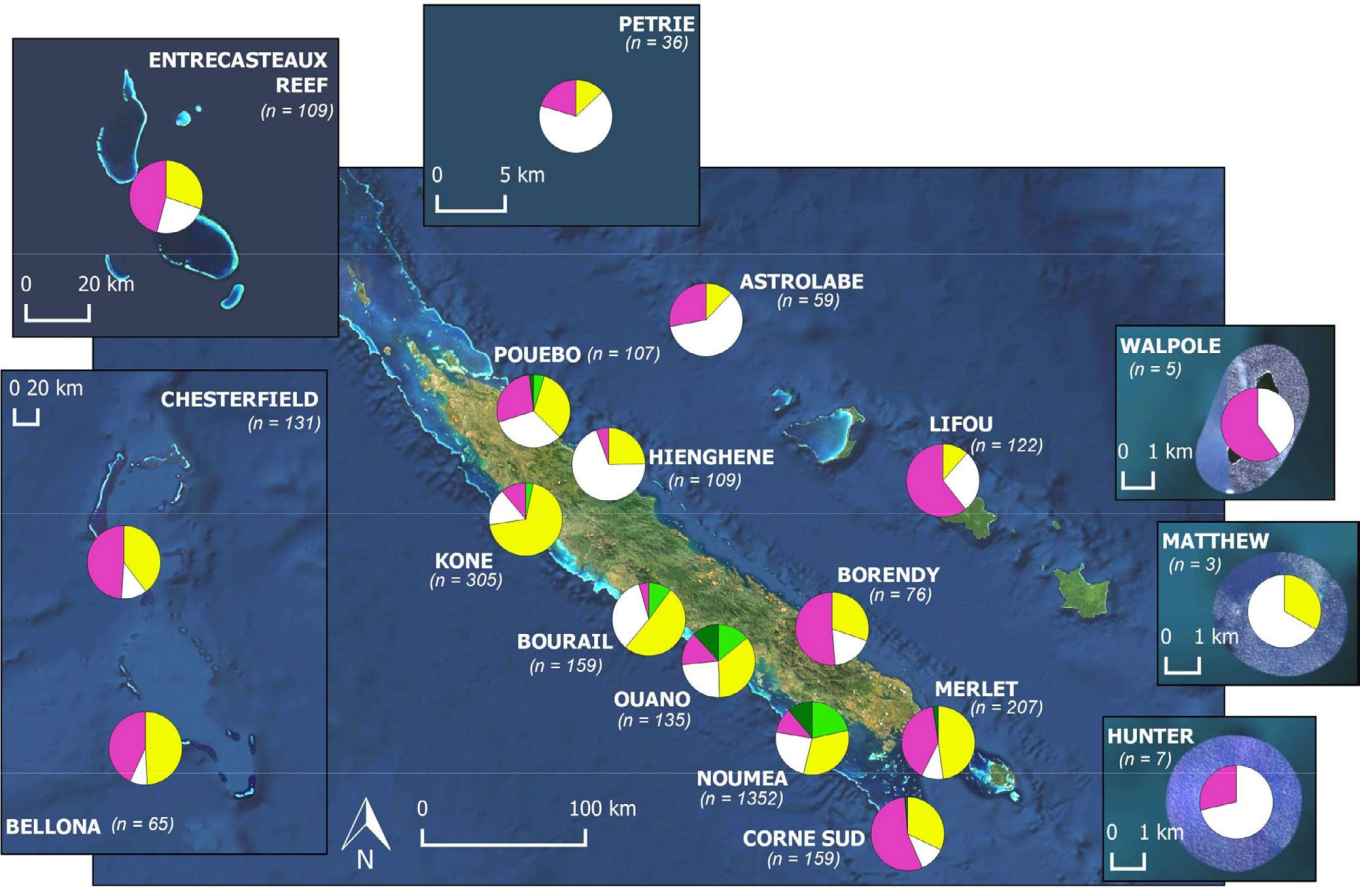
Distribution of the habitat proxy at each sampled site (number of observations in parentheses). Live Coral (pink), Debris (white), Sandy (yellow), Seagrass (light green), and Macroalgae (dark green)

The distribution of the habitat proxy across sites illustrated differences between sites (Figure 3, Appendix 5). Soft‐bottom habitats were more frequent on the western coast, consistently with a larger and shallower lagoon area. Hence, the prevalence of fringing seagrass beds was outstanding in Bourail (WH property) and macroalgae fields were common in Nouméa and Ouano areas. In contrast, stations in the Live Coral habitat were numerous at oceanic sites (48% at stations versus 17% at coastal stations; Figure 3, Appendix 4).

**FIGURE 4:**
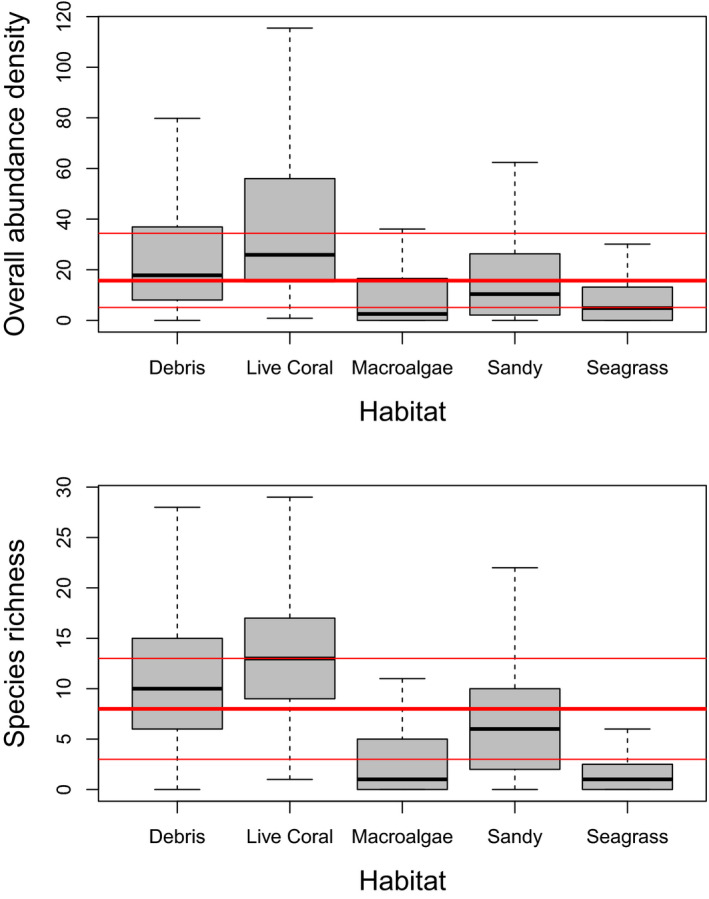
Overall abundance density (top, in ind/100 m^2^) and species richness (bottom, in number of species within a 5‐m radius around the camera) as a function of habitat proxy. Ends of boxes correspond to 0.25 and 0.75 quartiles, median as black line. 0.25, 0.5, and 0.75 quartiles for the same metrics computed over all habitats in red

**FIGURE 5:**
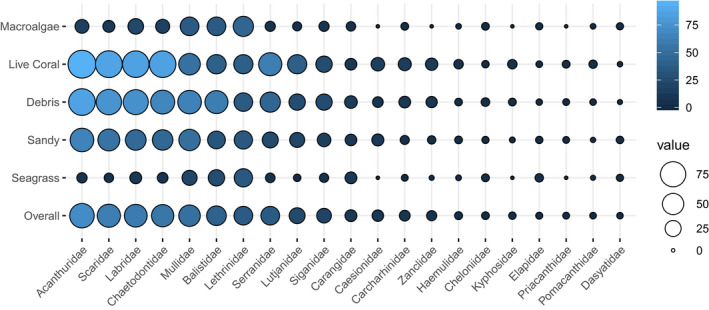
Frequency per family (% of stations where present) in each habitat (across sites)(circle size and color shade). Only families with overall frequency > 1% were reported (complete table of frequencies in Appendix 6)

The ability of the habitat proxy to explain variations in fish communities was first illustrated by comparing overall abundance density and species richness (SR) across habitats (Figure 4). Both metrics highly significantly varied across habitats (*p* < 2.2e−16, GLM with gamma and negative binomial distribution, respectively). Densities per habitat significantly differed from one another (Tukey's multiple comparisons, *p* <.01 ), except between Macroalgae and Seagrass habitats. SR per habitat all significantly and strongly differed from one another (Tukey's multiple comparisons, *p* < 1e−05). Community composition strikingly differed between habitats (Figure 5, Appendix 6). Four families (Acanthuridae, Scaridae, Labridae, and Chaetodontidae) dominated in the Live Coral, Debris, and Sandy habitats. In the Macroalgae and Seagrass habitats, Lethrinidae, Mullidae, Balistidae, and to a lesser extent Labridae were the most frequent families. Dasyatidae and Elapidae were mostly observed on soft bottoms, whereas Carcharinidae were mainly seen on hard bottoms, and rarely in Macroalgae and Seagrass habitats. Turtles were seen in all habitats.

### 3.2 Habitat heterogeneity explained through classification rules

For the Macroalgae habitat, four rules described 95% of stations with an 80% overall confidence (Table 3). This habitat being clearly characterized by erect algae, a lower threshold on algal turf cover was necessary to distinguish it from the Sandy habitat. Where algal cover was lower (MA4), a maximum value for seagrass cover discriminated this habitat from the Seagrass habitat. MA4 included stations displaying a mix of macroalgae, sandy, and seagrass covers.

**TABLE 3:** Mutually exclusive classification rules selected for soft‐bottom habitats. Rules were illustrated by screenshots in Pelletier (2020)

Habitat (No. of stations)	Classification rules	Conf. level (%)	No. of stations fulfilling the rule	Habitats for which confusion is possible (% stations)
Macroalgae (175)	MA1. Algae > 80% and algal turf < 20%	100	20	None
	MA2. 60% ≤ algae < 80% and algal turf ≤ 20% and dead coral < 5%	100	35	None
	MA3. 40% ≤ algae < 60% and algal turf < 20% and hard coral ≤ 20%	79	64	Sandy (7%) Sea grass beds (11%) Debris (2%)
	*MA4. 20% ≤ algae < 40% and algal turf < 5% and seagrass < 40% and hard coral < 5%*	*66*	*48*	*Sandy (25%)* *Seagrass beds (8%)* *Debris (1%)*
Seagrass (340)	SG1. Seagrass ≥ 80%	100	93	None
	SG2. 60% ≤ seagrass < 80%	98	100	Macroalgae (1%) Debris (1%)
	SG3. 40% ≤ seagrass < 60% and algae < 40%	93	92	Macroalgae (4%) Debris (2%) Sandy (1%)
	SG4. 20% ≤ seagrass < 40% and algae < 40% and depth ≥ 10	81	34	Macroalgae (12%) Debris (2%) Sandy (5%)
Sandy (1,157)	SA1. Sand ≥ 80% and seagrass < 20% and algae < 20%	99	393	Seagrass (1%)
	SA2. Sand ≥ 80% and seagrass < 20% and algae ≥ 20% and algal turf > 5%	97	69	Macroalgae (3%)
	SA3. 60% ≤ sand < 80% and seagrass < 5% and erect algae < 20%	97	417	Live Coral (2.5%) Debris (0.7%)
	SA4. 40% ≤ sand < 60% and 5 ≤ live coral < 40% and complexity < 2 and (debris + boulder + rock + slab) < 40%	87	88	Debris (8%) Live Coral (4%) Macroalgae (1%)
	*SA5. 40% ≤ sand < 60% and 5 ≤ live coral < 40% and 2* ≤ *complexity < 3 and (debris + boulder + rock + slab) < 40%*	*46*	*91*	*Live Coral (33%)* *Debris (21%)*

Note

“Algae” cover corresponds to the sum of “erect algae” and “algal turf” covers. Rules with a confidence lower than 70% are in italics.

In the Seagrass habitat, four rules described 94% of stations with a 95% overall confidence. 58% of stations (SG1 and SG2) comprised stations with dense and healthy seagrass beds. SG3 corresponded to a mix of seagrass and macroalgae. Where seagrass cover was lower (SG4), a third condition was needed to discriminate this habitat from Macroalgae, Debris, and Sandy habitats. SG4 corresponded to deeper areas with sparser seagrass beds.

In the case of Sandy habitat, 84% of stations were captured from four rules with a 96% overall confidence. Where sand cover ≥ 60%, two more conditions avoided confusion with the Seagrass and Macroalgae habitats (SA1 to SA3), while for lower sand covers, additional conditions prevented confusion with the hard‐bottom habitats (SA4). For sand covers below 60%, SA5 captured 91 stations, but confidence dropped due to confusion with the Live Coral and Debris habitats, and no additional condition could be determined to improve confidence. Likewise, no rule with sufficient confidence could be identified for the 22 stations with a sand cover ranging between 20% and 40%, these resembling stations from the Live Coral and Debris habitats.

For Live Coral habitat, six rules described 90% of stations with a 92% overall confidence (Table 4). LC1 indicated a good or excellent status of coral cover. Where live coral cover was lower, a second condition on hard coral cover was necessary (LC2 to LC5). A hard coral cover ≥ 60% clearly distinguished the Live Coral habitat from the Debris habitat where the dominant substrate was a mixture of debris, boulders, rock, and slab. These rules (LC2 and LC4) corresponded to high hard coral cover but medium live coral cover, pointing to a not‐so‐good status of coral cover. Where hard coral cover < 60%, possible confusion with the Debris and Sandy habitats increased (LC3). If in addition live coral cover was lower (LC5), confusion with Debris and Sandy habitats was reduced by additional conditions on debris‐related cover, and minimum complexity and topography. LC5 illustrated the fact that complexity was higher in the Live Coral habitat than in the Debris habitat. At even lower live coral covers (<20%), confusion with the Debris and Sandy habitats was minimized by adding two conditions (LC6) that are—interestingly—linked to a poor status of live coral (presence of algal turf and substantial dead coral cover).

**TABLE 4:** Mutually exclusive classification rules selected for hard substrate habitats. Rules were illustrated by screenshots in Pelletier (2020)

Habitat (No. of stations)	Classification rules	Conf. level (%)	No. of stations fulfilling the rule	Habitats for which confusion is possible (% stations)
Live Coral (717)	LC1: Live coral ≥ 60%	100	217	None
	LC2: 40% ≤ live coral < 60% and hard coral ≥ 60%	100	121	None
	LC3: 40% ≤ live coral < 60% and hard coral < 60%	73	61	Debris (15%) Sandy (12%)
	LC4: 20% ≤ live coral < 40% and hard coral ≥ 60%	99	105	Debris (1%)
	LC5: 20% ≤ live coral < 40% and hard coral < 60% and (debris + boulder + rock + slab) < 40% and complexity > 2 and Topography > 2	86	108	Sandy (11%) Debris (3%)
	LC6: 15% ≤ live coral < 20% and dead coral ≥ 20% and algal turf ≥ 5%	89	41	Sandy (7%) Debris (4%)
Debris (756)	D1. Debris ≥ 60%	100	107	None
	D2. 40% ≤ debris < 60% and boulder ≥ 1%	100	98	None
	D3: 40% ≤ debris < 60% and boulder < 1% and sand < 40%	89	42	Live Coral (11%)
	D4: 40% ≤ debris < 60% and boulder < 1% and sand ≥ 40% and complexity > 1.5	75	6	Sandy (12.5%) Macroalgae (12.5%)
	D5: 20% ≤ debris < 40% and sand < 60% and (slab + boulder + rock) ≥ 20	97	113	Live Coral (3%)
	D6: 20% ≤ debris < 40% and sand < 60% and 5 < (slab + boulder + rock) < 20 and complexity > 2	87	71	Live Coral (12%) Sandy (1%)
	D7: 5% ≤ debris < 20% and (slab + boulder + rock) ≥ 20 and sand < 60% and hard coral < 40%	87	103	Live Coral (7%) Sandy (9%)

In the Debris habitat, seven rules described 72% of stations with a 93% overall confidence level. This habitat was assigned with high confidence for either of the following single conditions (only D1 was selected in Table4 as the other rules had low support): debris cover ≥ 60% (D1, conf. = 100%), slab cover ≥ 60% (conf. = 96%), boulder cover ≥ 40% (conf. = 86%), and rock cover ≥ 20% (conf. = 97%). Each of these conditions described facies (defined here as a set of morphological, physical, and biological features) that were only or mostly found in this habitat. Rules involving a composite variable corresponding to the sum of other debris‐related variables were selected as they have a larger support. Small (D1) or intermediate (D2) substrate granularity was the most frequent patterns. For D3 to D7, more conditions were required to discriminate Debris habitat from Sandy, Macroalgae, and Live Coral habitats; and for debris cover < 40%, a condition on the composite debris‐related cover was needed. D5 and D7 corresponded to a higher substrate granularity (including slab and rock). The Debris habitat was the most heterogeneous.

The Debris habitat was the most heterogeneous. Screenshots of frames from video footages illustrated each rule (Pelletier, 2020).

### 3.3 Habitat prediction from rules

Based on the 1,000 rules obtained from the TopK algorithm, habitat was predicted correctly for 70% to 84% of the stations depending on the required confidence level (Table 5, column 5). However, the Macroalgae habitat could not be predicted at all (columns 2–4), because with fewer stations, it was described by rules with smaller supports that were not among the 1,000 rules with maximum support.

**TABLE 5:** Proportion of stations classified and corresponding confidence for the (a) Top1000 rules with three conditions (columns 2–4), and (b) rules from Tables 3 and 4 (columns 6–7)

Habitat	Top1000 rules CL				Expert‐selected rules (26)	
	80%	90%	95%	Number of rules needed at 95% CL	% stations classified	Overall CL
Macroalgae	0	0	0	‐	95	80
Seagrass	75	76	80	221	94	95
Sandy	91	82	73	83	91	88
Live Coral	91	84	80	494	91	93
Debris	90	82	70	202	72	93
Overall	84	77	70	1,000	87	91

Abbreviation: CL, confidence level.

The 26 expert‐selected rules (Tables 3 and 4) may be used instead for prediction (Table 5, columns 6 and 7). In the Macroalgae habitat, 95% of stations were thus predicted with an overall confidence of 80%. Confidence was 100% where algal cover exceeded 60% (MA1 and MA2), and 80% for covers below 60% (MA3 and MA4). The Seagrass habitat was well predicted from the rules (94% of stations with a 95% overall confidence) with a low probability of confusion.

In the Sandy habitat, 91% of the stations were predicted with an 88% overall confidence. Probability of confusion was <3% for SA1 to SA3, increased for SA4 and mostly for SA5. Habitat was not reliably predicted only for 2% of stations in this habitat (corresponding to 20% < sand cover ≤ 40%).

In the Live Coral habitat, 90% of stations were correctly classified from six rules encompassing the range of live coral covers observed (overall confidence 92%). Even at low live coral covers, habitat prediction had a high confidence and a low probability of confusion with the Sandy and Debris habitats.

In contrast, only 72% of stations in the Debris habitat were correctly classified from seven rules (overall confidence 93%). Additional rules of increasing complexity would be necessary to classify the remaining 28% of stations. In general, where live coral cover or debris cover comprised between 5% and 40%, stations could belong to three habitats: Live Coral, Debris, or Sandy.

Overall, the 26 rules selected enabled more stations to be correctly classified in each habitat, and with higher confidence than the Top1000 rules.

## 4 DISCUSSION AND PERSPECTIVES

### 4.1 A regionally scalable habitat proxy for consistent assessments

We have developed a methodology to construct a habitat classification that we applied to a large data set of sampling stations distributed over the entire reef and lagoon areas of NC (1.4 million km^2^) and with a systematic coverage in all major habitats. Habitat attributes are summarized into five habitat types, which are consistent across spatial scales, and represent a satisfactory compromise between parsimony and relevance for assessment and management. Three are essential habitats for coral reef ecosystems (Seagrass, Macroalgae, and Live Coral). Three correspond to soft‐bottom areas rarely surveyed (Seagrass, Macroalgae, and Sandy).

Variations in overall abundance and species richness were highly significantly related to habitat type (i.e., the proxy) despite the variety of settings encompassed in our large data set. This established the importance of the habitat proxy (covariate) for assessing fish communities. Family dominance differed according to habitat; several families including important fished species, for example, Lethrinidae, are more frequent in soft‐bottom habitats. Although diversity and abundance were highest in reef areas stricto sensu (i.e., hard bottoms), soft‐bottom habitats host a number of species belonging to many families and in substantial abundances. Simultaneous in situ measurements of fish and habitat covering both hard and soft bottoms enable cross‐habitat comparisons of important metrics such as fish abundance and species richness. Also, they enable monitoring changes in habitat that affect fish stock status (Brown et al., 2019). Studies using simultaneous in situ measurements of fish and habitat covering both hard and soft bottoms are few. Ricart, Sanmartí, Pérez, and Romero (2018) collected fish data from Underwater Visual Censuses and habitat data from video transects. Small‐scale habitats were directly identified by visual analysis and directly classified into seagrass, rocky reefs, and sand, to compare fish‐related metrics across habitats along a 100‐km stretch of coast in the NW Mediterranean. Yates, Mellin, Caley, Radford, and Meeuwig (2016) used baited video for surveying fish, and both video (baited and towed) and remote sensing to survey habitat in an area of 200 km^2^ encompassing a wide range of subtropical habitats. Habitats were categorized either visually according to habitat complexity (for baited video) or by using the CATAMI classification scheme (Althaus et al., 2015) (for towed video). But habitat descriptors were not integrated into a habitat typology.

Overall, our results illustrated the strong dependence of fishes upon very small‐scale (<100 m^2^) habitat features, both biotic and abiotic (see references in §1), and the feasibility of a scalable approach. The habitat proxy was successfully used in assessments of benthic habitats and fish communities in NC (see §4.4).

Owing to the spatial coverage of the data, general patterns of habitat distribution in vast reef and lagoon areas were for the first time evidenced from comprehensive field measurements covering the entire EEZ of New Caledonia: the prevalence of Seagrass and Macroalgae habitats in the western lagoon of the main island and, importantly, high live coral covers frequently observed at oceanic reefs remote from anthropogenic pressures.

We are not aware of any study characterizing coastal habitats from direct measurements encompassing all habitats over such vast management units. Existing studies either pertained to smaller areas (e.g., Davis, Harasti, & Smith, 2016), relied on remote‐sensing data (Mellin et al. (2012), or focused on particular habitats (Curley, Kingsford, & Gillanders, 2003; Setyawidati et al., 2018).

### 4.2 Classification rules for habitat description and prediction

Supervised classification rules constitute a novel approach to habitat description previously achieved through clustering (Pelletier et al., 2012), nonparametric multidimensional scaling (Davis et al., 2016; Giménez‐Casalduero, Gomariz‐Castillo, & Calvín, 2011), or other statistical modeling.

In cluster analysis, within‐habitat heterogeneity is measured through the variance of habitat attributes in each cluster. In contrast, classification rules tackle heterogeneities through distinct sets of conditions on threshold values for habitat attributes. In this respect, rules capture both local heterogeneities and nonlinearities, representing an original and complementary approach for pattern analysis in large multivariate data sets.

The 26 selected rules described the main facies encountered in each habitat based on biotic (seagrass, live coral, erect macroalgae, and algal turf) and hard substrate covers. At medium or low values of these attributes, decreased confidence and possible confusions indicated a continuum between habitats. The rules confirmed the relevance of the typology and provided a refined and easy‐to‐grasp habitat description. Furthermore, the ruleset predicted habitat for 87% of stations in all habitats with an overall confidence ≥ 91%.

Rule selection was made possible by including expert knowledge, for example, considering archetypical habitat attributes, combining rules, using additional attributes (algal turf), and deriving relevant and mutually exclusive rules. Supervised classification rules belong to data mining—Knowledge Discovery from Data (KDD)—the process of discovering patterns in data (Witten & Frank, 2005). Inputs from domain (here ecology) experts are an important and acknowledged component of KDD because results produced by algorithms are overly numerous and must be further analyzed to unravel meaningful patterns. Adamo et al. (2016) combined expert rules and earth observation data to map wetland habitats. Our results illustrate the importance of embracing expert knowledge within workflows for large data sets.

### 4.3 Implications for conservation and management

The habitat proxy was derived from a comprehensive baseline data set comprising areas subject to a range of anthropogenic pressures, and it is consistent at the scale of New Caledonia's EEZ, including the 1.3 million km^2^ CSMP and the 15,743 km^2^ WH property. It has been successfully used in a number of assessments of the ecological status of fish communities, biotic covers, and other marine animals such as turtles (e.g., Pelletier, Bockel, Roman, Carpentier, & Laugier, 2016; Pelletier et al., 2014; Schohn, Bockel, Carpentier, & Pelletier, 2017; Schohn, Pelletier, & Carpentier, 2017), where it better explained habitat‐related variations of biodiversity than geomorphological maps.

The abundance, diversity, and community composition observed in the five habitats showed that an ecosystem‐based monitoring strategy must encompass not only reef areas (hard‐bottom areas) but also soft‐bottom areas, such as in this case Sandy, Seagrass, and Macroalgae habitats. Designing future surveys will benefit from our results, and the ruleset will be used to predict habitat with high confidence for any new observation.

Both the rules and the habitat proxy are thus useful tools for monitoring‐based assessment of habitat and associated macrofauna.

The study relies on an underwater video technique, which simultaneously records benthic habitat and fishes at the same exact spatial scale, and at a relatively low cost per observation: (a) 4–6 observations collected per field hour with two systems; (b) no expert needed on the field; (c) image postprocessing: 15–20' for habitat and 45–90' for fish (per footage); and (d) one system approximately costs 4,500 €. Pelletier et al. (2012) compared the cost‐effectiveness of Underwater Visual Censuses and STAVIRO techniques. Recent work in New Caledonia showed that on average one STAVIRO station requires 4 hr 30 min of work from fieldwork to assessment production (D. Pelletier, unpubl. data); however, the comparison with other techniques must account for the fact that many more observations are produced by STAVIRO for a given sampling effort on the field. Training in image analysis is achieving by joint analysis with a trained observer and requires 1 month on average. Identification skills are progressively gained, uncertainties being systematically checked by experts based on screenshots or video clips. The same protocol was implemented in temperate ecosystems (~900 valid footages in the Mediterranean and in the northeast Atlantic; D. Pelletier, unpubl. data) with lower underwater visibility, showing the relevance of the STAVIRO to various environments (see also Donaldson et al. (2019) for image postprocessing solutions to handle poor visibility). These data are currently being analyzed for assessments of habitats and fish communities.

This modeling approach may apply to any data set aimed at characterizing and predicting local habitat for assessments at larger scales. More generally, it could apply to other data sets where an observation is described by a number of attributes, for example, habitat attributes or species presence or abundance, obtained from other observation protocols.

As the numbers and sizes of monitoring data sets grow, robust data analysis tools and methods are needed to (a) update knowledge base as monitoring is conducted; (b) summarize numerous ecological attributes into a tractable nontechnical description; and (c) use these synthetic descriptions in assessments. Easy‐to‐understand descriptions, ideally complemented by in situ images and maps (Appendix 5), support the uptake of outcomes by scientists, by managers, and by a broader audience. Complementary efforts to develop interfaces that facilitate knowledge uptake by end users are underway.

## CONFLICT OF INTEREST

None declared.

## AUTHOR CONTRIBUTIONS

**Dominique Pelletier:** Conceptualization (lead); Data curation (supporting); Formal analysis (lead); Funding acquisition (lead); Investigation (lead); Methodology (lead); Project administration (lead); Resources (equal); Software (equal); Supervision (lead); Validation (equal); Visualization (supporting); Writing‐original draft (lead); Writing‐review & editing (lead). **Nazha Selmaoui‐Folcher:** Conceptualization (supporting); Formal analysis (equal); Investigation (supporting); Methodology (equal); Resources (supporting); Software (equal); Writing‐review & editing (equal). **Thomas Bockel:** Data curation (equal); Formal analysis (equal); Investigation (equal); Methodology (equal); Software (equal); Writing‐review & editing (equal). **Thomas Schohn:** Data curation (equal); Formal analysis (equal); Investigation (equal); Methodology (equal); Software (equal); Visualization (lead); Writing‐review & editing (equal).

## Figures and Tables

**TABLE A4:**
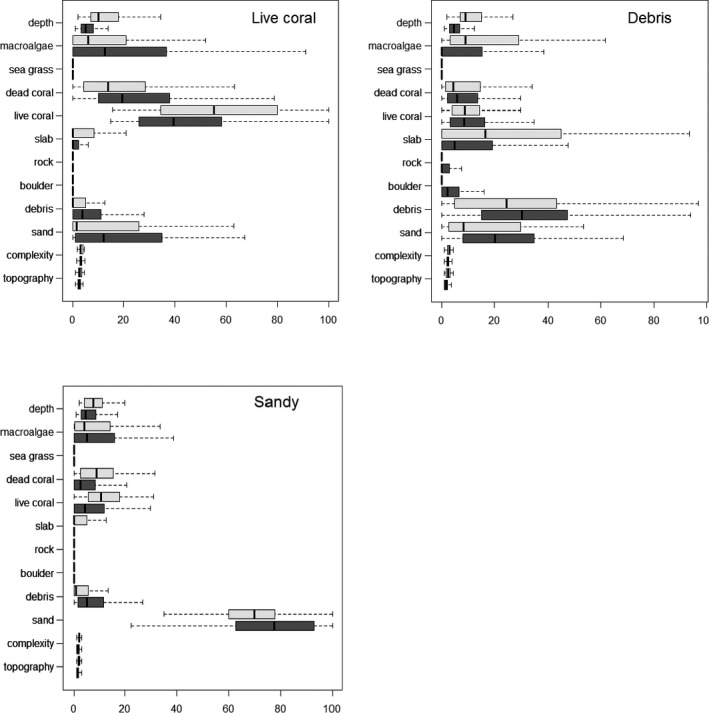
Boxplots of habitat attributes for the three habitats encountered at both coastal and oceanic stations: light grey: CSMP (oceanic) stations; dark grey: coastal stations

Note

For each boxplot, whiskers extend to 1.5 *box length away from each side of the box (default setting in R); therefore, values above or below these are not plotted (for sake of readability).

## Data Availability

Data are accessible at https://doi.org/10.12770/4380a3ad‐0f6d‐41ec‐8a84‐8ee3bb227e35.

## Appendix 1

**TABLE A1:** List of taxonomic families considered in image analysis and in the metrics reported in Figure 4

Acanthuridae	Lutjanidae
Albulidae	Malacanthidae
Aulostomidae	Megalopidae
Balistidae	Mugilidae
Caesionidae	Mullidae
Carangidae	Polynemidae
Carcharhinidae	Pomacanthidae
Chaetodontidae	Priacanthidae
Chanidae	Rhincodontidae
Chirocentridae	Rhinobatidae
Dasyatidae	Scaridae
Diodontidae	Scombridae
Ephippidae	Serranidae
Gerreidae	Siganidae
Ginglymostomatidae	Sphyraenidae
Fistulariidae	Stegostomatidae
Haemulidae	Tetraodontidae
Kyphosidae	Zanclidae
Labridae	*Chelonidae*
Lamnidae	*Dugongidae*
Leiognathidae	*Elapidae*
Lethrinidae	

Note

The 39 fish families include all fished species and iconic species, which are identifiable within a 5 m distance under the observation protocol. Hence, small (in practice, Lmax < 18 cm), cryptic, and nocturnal species are excluded from the list. Three families of conspicuous iconic nonfish species (in italics), namely marine turtles, sea snakes, and dugongs, are also included.

## Appendix 2

### Method details for the construction of the habitat typology

The clusters were first obtained through a combination of principal component analysis (PCA) and hierarchical ascending clustering (HAC) (Pelletier & Ferraris, 2000). The number of clusters was determined from Ward's (1963) criterion based on a trade‐off between relevance and parsimony.

Clusters were then checked for stations not assigned to the most relevant cluster, which may occur in unsupervised techniques. Hence, in each cluster characterized by an archetypical biotic cover, stations with this cover less than 15% were set aside. 15% corresponded to the presence of the biotic cover on a single frame of the station and was a reasonable expert‐based threshold. A random forest (RF) algorithm (Breiman, 2001) was trained from the other coastal stations and then used to predict cluster (i.e. habitat proxy) for each station set aside, enabling to reclassify the station in a more appropriate cluster with a known confidence level.

To assign a habitat to the 536 remote stations, a second RF model was then trained from the typology of coastal stations and used to predict habitat at these remote stations.

Resulting clusters were characterized by habitat descriptors by testing differences in means between each cluster and the overall set of stations (Pelletier & Ferraris, 2000). Analyses were performed with R 3.5.1 (R Core Team, 2018) using the FactomineR package (V 1.41, Lê, Josse, & Husson, 2008) and the randomForest package (Liaw & Wiener, 2002).

## Appendix 3

### Method details for classification rules

#### What are classification rules?

Association rules are used to describe multivariate data sets, particularly for mining large data sets of categorical variables (Agrawal et al., 1993). An association rule r is an implication of the form r: **R** →**Q**, with **R** the antecedent of the rule and **Q** the consequent of the rule. Classification rules are association rules that conclude to a particular attribute being a label, for example, a class index. The label of the consequent **Q** was here the habitat proxy from the typology, while the antecedent **R** comprised the conditions on the habitat descriptors.

#### Additional constraints for the supervised classification algorithm

A huge number of rules may be result from the combinatory of conditions on categorical variables. Constraints are thus set to discover the most interesting and relevant rules (McGarry, 2005). Objective constraints are interestingness measures (Freitas, 1999) or statistical measures, while subjective constraints are often formulated by domain experts. Three objective constraints were considered here: (a) the number of conditions on habitat descriptors was ≤3; (b) the support, and (c) confidence (Table A2). We used the TopKRules algorithm (Fournier‐Viger et al., 2012), which extracts rules with a confidence larger than a minimum threshold (min_conf), and retains the K ones with maximum support. TopK rules were searched for K = 1,000, and for three min_conf values, 80%, 90%, and 95%, producing three sets of 1,000 rules. In each set, a given station may satisfy several rules, and conversely, some stations may not satisfy any rule, for example, if they correspond to rare patterns.

For the TopK algorithm, quantitative habitat descriptors were recoded as categorical variables based on bins of equal width, except for extreme values, which may be meaningful for characterization. Cover categories were <5%, [5%,20%[, [20%,40%[, [40%,60%[, [60%,80%[, [80%,95%[, and ≥95%. Depth categories were [0.8 m,5 m[, [5 m,10 m[, [10 m,15 m[, [15 m,20 m[, [20 m,25 m[, and ≥25 m. Topography and complexity were recoded in four categories: [1–2[, [2–3[, [3–4[, and [4–5[. The TopKRules algorithm was implemented using the SPMF software (Fournier‐Viger et al., 2014).

#### Expert‐based constraints to select among the rules

The 1,000 rules found by the algorithm were selected and reorganized, in order to achieve a compromise between representativeness (i.e., a large proportion of the stations in each habitat were described by the rules with a high confidence level) and parsimony (not having too many rules). In addition to the constraints on support and confidence, the following constraints were thus set for each rule: (a) include a condition on the archetypical (also termed paragon) attribute of each habitat; (b) comprise up to four conditions on habitat attributes; and (c) not overlap with another rule, the set of rules then formed a partition of the stations in each habitat and over all habitats.

A large support meant the rule described a frequent pattern, and this was desirable since we aimed at identifying rules accounting for as many stations as possible in each habitat. A large confidence indicated that the rule would reliably predict habitat from habitat descriptors, which was also an objective of the analysis.

Specific habitat attributes not considered in the typology were included in the rules to increase confidence when they were deemed relevant to describe within‐habitat heterogeneity. In some habitats, rules with a lower confidence were useful to increase support and gain more explanation about within‐habitat heterogeneity. The resulting set of rules was then used to describe this heterogeneity.

#### Habitat prediction from classification rules

In the case of classification rules, the set of solutions forms a classification model ordered by decreasing support and confidence. This model was used to predict the label of a new individual by finding the first rule it satisfies within the set of solutions. We determined the ability of both the Top1000 and the expert‐selected rules to predict habitat with a satisfactory confidence level.

**TABLE A2:** Algorithm settings for the supervised classification algorithm used (TopKRules)

Parameter	Definition	Relevance to the study's objective
Number of conditions	Number of conditions in the subset R	A simple rule is preferred. But more complex rules may be needed to assign more stations to clusters
K	Number of rules r to be searched for	More rules enable to assign more rules to clusters
Confidence	Proportion of stations that are correctly assigned to the cluster based on the rule, i.e. Card(r)/Card(R))	A high confidence level is needed to classify stations correctly. In return, particular (and thus) rare stations may be assigned with a lower confidence level
Support	Number of individuals satisfying the rule (potentially not belonging to the cluster if confidence level is smaller than one)	Rules with a larger support are preferred as they correspond to more frequently encountered conditions. However, particular conditions also occur in relation to specific features of habitat

Note

Note that the higher the confidence, the smaller the rule's supports, meaning a trade‐off between support and confidence.

## Appendix 4

### Statistics and boxplots for habitat attributes

**TABLE A3:** Mean and standard deviation of habitat attributes in each habitat, with numbers of stations in parentheses

	Overall	Seagrass (340)	Macroalgae (175)	Sandy (1,157)	Sandy (144)	Debris (755)	Debris (134)	Live coral (714)	Live coral (254)
	All	All	All	All	Oceanic	All	Oceanic	All	Oceanic
Topography	1.8 ± 0.9	1.1 ± 0.3	1.2 ± 0.4	1.5 ± 0.5	1.9 ± 0.5	1.9 ± 0.8	2.5 ± 0.9	2.7 ± 0.7	2.8 ± 0.9
Complexity	2.2 ± 0.8	1.3 ± 0.5	1.7 ± 0.7	1.7 ± 0.6	2.1 ± 0.5	2.5 ± 0.7	2.8 ± 0.8	3.2 ± 0.6	3.2 ± 0.6
Sand (%)	52.3 ± 29.4	96.3 ± 5.9	91.7 ± 9.1	**75.6 ± 17.3**	**69.0 ± 13.0**	21.1 ± 16.3	16.4 ± 16.4	17.1 ± 18.8	13.6 ± 18.4
Debris (%)	12.3 ± 18.5	2.5 ± 4.2	6.2 ± 7.6	7.5 ± 8.3	4.1 ± 6.4	**31.6 ± 23.1**	**28.9 ± 27.5**	5.9 ± 8.6	3.5 ± 6.7
Boulder (%)	1.5 ± 4.9	0.1 ± 0.5	0.3 ± 0.8	0.6 ± 1.8	0.4 ± 1.8	**4.6 ± 8.5**	**2.9 ± 8.8**	0.5 ± 2.2	0.3 ± 2.3
Rock (%)	0.9 ± 7.7	0.0 ± 0.2	0.0 ± 0.2	0.3 ± 1.5	0.1 ± 0.7	**3.2 ± 9.4**	**2.8 ± 15.2**	0.2 ± 1.5	0.0 ± 0.0
Slab (%)	5.2 ± 18.8	0.1 ± 0.8	0.3 ± 1.5	1.4 ± 4.0	3.5 ± 6.2	**15.1 ± 20.5**	**26.2 ± 27.8**	4.4 ± 9.4	6.4 ± 11.9
Live coral (%)	16.9 ± 29.9	0.7 ± 2.0	0.8 ± 2.2	8.2 ± 9.3	12.2 ± 8.5	11.3 ± 10.3	10.4 ± 8.8	**48.6 ± 24.0**	**57.5 ± 26.1**
Dead coral (%)	10.8 ± 16.2	0.3 ± 0.9	0.3 ± 1.1	6.4 ± 8.6	10.6 ± 9.6	13.0 ± 19.4	12.4 ± 17.8	23.3 ± 20.1	18.3 ± 17.5
Sea grass (%)	8.1 ± 0.5	**63.4 ± 21.9**	10.9 ± 14.6	1.4 ± 4.6	0.1 ± 1.0	0.4 ± 3.9	0.0 ± 0.0	0.0 ± 0.3	0.0 ± 0.0
Macroalgae (%)	15.8 ± 18.0	8.3 ± 11.3	**52.0 ± 19.5**	11.0 ± 16.1	8.8 ± 11.2	15.4 ± 25.5	18.8 ± 22.5	18.7 ± 22.0	13.8 ± 17.7
Depth (m)	7.6 ± 9.0	9.0 ± 4.9	9.2 ± 5.3	6.7 ± 5.2	9.1 ± 6.7	6.8 ± 6.0	13.3 ± 10.3	8.7 ± 7.0	13.5 ± 8.9

Note

Values for remote oceanic stations shown separately for habitats found at these locations. Values for archetypical attributes in each habitat are in bold.

## Appendix 5

### Results at site scale: number of stations per habitat and habitat maps

On following maps, orange lines delineate the WH property, green lines delineate marine protected areas. GIS layers may be accessed on a map server (Schohn & Pelletier, 2018)

**TABLE A5:** Number of stations per habitat in each site

Site	Macroalgae	Live coral	Debris	Sandy	Seagrass
Astrolabe	0	28	24	7	0
Bellona	0	28	5	32	0
Bourail	0	7	55	81	16
Borendy	0	39	14	23	0
Chesterfield	0	64	15	52	0
Corne Sud	2	88	18	51	0
Entrecasteaux	0	50	26	33	0
Noumea	150	152	322	438	290
Hienghène	0	6	76	27	0
Hunter	0	2	5	0	0
Kone	0	34	50	212	10
Lifou	0	74	34	14	0
Matthew	0	0	2	1	0
Merlet	5	83	20	99	0
Ouano	16	20	32	48	19
Petrie	0	9	21	4	0
Pouebo	2	30	35	35	5
Walpole	0	3	2	0	0

Note

See Figure 1 for site location.

## Appendix 6

**TABLE A6:** Frequency per family in each habitat of the typology

	Macroalgae	Live Coral	Debris	Sandy	Seagrass	*Overall*
Acanthuridae	16.9	**94.6**	**85.3**	**64.5**	7.2	*70.3*
Scaridae	11.3	**86.3**	**76**	**53.7**	5.5	*61.5*
Labridae	23.2	**84.4**	**73.8**	**46.7**	11	*59*
Chaetodontidae	17.6	**84.6**	**66.5**	**45.9**	8.1	*56.2*
Mullidae	36.6	**51.8**	**64**	**50.7**	21.2	*51.5*
Balistidae	**36.6**	**40**	**60.5**	32.1	26.3	*41.5*
Lethrinidae	**44.4**	**39.1**	**36**	34.6	34.7	*36.6*
Serranidae	7	**61.3**	**44.6**	**24.9**	5.9	*36.5*
Lutjanidae	4.9	**39.1**	**26.2**	**21**	2.5	*24.4*
Siganidae	7	**26.3**	**25.8**	**16**	5.1	*19.8*
Carangidae	5.6	**10.7**	**13.3**	10.4	**11**	*11.1*
Caesionidae	0	**15.9**	8	**11.5**	0	*10.1*
Carcharhinidae	2.8	**14.3**	**10.7**	**4.9**	1.7	*8.4*
Zanclidae	0	**12.4**	**9.5**	**3.7**	0.4	*6.9*
Haemulidae	0.7	**5.6**	**2.7**	**2.8**	0.8	*3.1*
*Cheloniidae*	**2.8**	2.4	**4.1**	2.1	**3**	*2.8*
Kyphosidae	0	**5.6**	**2.9**	**0.9**	0	*2.4*
*Elapidae*	**2.1**	1.5	1.3	**2.3**	**3.8**	*1.9*
Priacanthidae	0	**2.9**	**1.7**	**1.7**	0	*1.7*
Pomacanthidae	**0.7**	**3.7**	**1.4**	0.5	0.4	*1.5*
Dasyatidae	**2.1**	0.6	0.4	**2.5**	**2.1**	*1.4*
Aulostomidae	0	**3.4**	**0.1**	**0.2**	0	*0.9*
Tetraodontidae	**0.7**	**1**	**1**	0.4	0.4	*0.7*
Scombridae	**1.4**	0.6	**0.8**	0.5	**0.8**	*0.7*
Diodontidae	0	**0.7**	**0.4**	**0.6**	**0.4**	*0.5*
Ephippidae	**0.7**	0.3	**0.6**	0.1	**1.7**	*0.5*
Myliobatidae	**0.7**	0.1	**0.6**	**0.2**	0	*0.3*
Chanidae	**1.4**	0	0.1	**0.4**	**0.4**	*0.3*
Sphyraenidae	0	**0.3**	**0.3**	**0.4**	0	*0.3*
Ginglymostomatidae	0	**0.3**	**0.1**	0	0	*0.1*
Stegostomatidae	0	**0.1**	0	**0.2**	0	*0.1*
Mugilidae	0	**0.1**	0	0	**0.4**	*0.1*

Note

Families are ordered by decreasing overall frequency (frequency computed over all habitats). For each family, the three habitats with maximum frequency are in bold.
